# Quenching of quorum sensing in multi-drug resistant *Pseudomonas aeruginosa*: insights on halo-bacterial metabolites and gamma irradiation as channels inhibitors

**DOI:** 10.1186/s12941-024-00684-5

**Published:** 2024-04-10

**Authors:** Reham Talaat, Mohamed N. Abu El-naga, Heba Abd Alla El-Bialy, Mohie Z. El-Fouly, Mohamed A. Abouzeid

**Affiliations:** 1 Radiation Microbiology Department, National Center for Radiation Research and Technology (NCRRT), Egyptian Atomic Energy Authority (EAEA), Cairo, Egypt; 2 Microbiology Department, Faculty of Science, Ain Shams University, Cairo, Egypt; 3 Faculty of Science, Galala University, Suez, Egypt

**Keywords:** Quorum sensing, *Pseudomonas aeruginosa* strains, Gamma irradiation, Bio-active metabolites, Multi-drug resistance

## Abstract

**Background:**

Anti-virulence therapy is a promising strategy to treat multi-drug resistant (MDR) pathogens. *Pseudomonas aeruginosa* is a potent opportunistic pathogen because of an array of virulence factors that are regulated by quorum sensing systems.

**Methods:**

The virulence features of four multi-drug resistant *P. aeruginosa* strains were investigated upon exposure to the sub-lethal dose of gamma rays (1 kGy), and sub-inhibitory concentrations of bioactive metabolites recovered from local halophilic strains in comparison to control. Then, the gene expression of AHL-mediated quorum sensing systems (las/rhl) was quantitatively determined in treated and untreated groups by real-time PCR.

**Results:**

The bioactive metabolites recovered from halophilic strains previously isolated from saline ecosystems were identified as *Halomonas cupida* (Halo-Rt1), *H. elongate* (Halo-Rt2), *Vigibacillus natechei* (Halo-Rt3), *Sediminibacillus terrae* (Halo-Rt4) and *H. almeriensis* (Halo-Rt5). Results revealed that both gamma irradiation and bioactive metabolites significantly reduced the virulence factors of the tested MDR strains. The bioactive metabolites showed a maximum efficiency for inhibiting biofilm formation and rhamnolipids production whereas the gamma irradiation succeeded in decreasing other virulence factors to lower levels in comparison to control. Quantitative-PCR results showed that AHL-mediated quorum sensing systems (las/rhl) in *P. aeruginosa* strains were downregulated either by halo-bacterial metabolites or gamma irradiation in all treatments except the upregulation of both lasI internal gene and rhlR intact gene in *P. aeruginosa* NCR-RT3 and both rhlI internal gene and rhlR intact gene in *P. aeruginosa* U3 by nearly two folds or more upon exposure to gamma irradiation. The most potent result was observed in the expression of lasI internal gene that was downregulated by more than ninety folds in *P. aeruginosa* NCR-RT2 after treatment with metabolites of *S. terrae* (Halo-Rt4). Analyzing metabolites recovered from *H. cupida* (Halo-Rt1) and *H. elongate* (Halo-Rt2) using LC–ESI–MS/MS revealed many chemical compounds that have quorum quenching properties including glabrol, 5,8-dimethoxyquinoline-2-carbaldehyde, linoleoyl ethanolamide, agelasine, penigequinolones derivatives, berberine, tetracosanoic acid, and liquidambaric lactone in the former halophile and phloretin, lycoctonine, fucoxanthin, and crassicauline A in the latter one.

**Conclusion:**

QS inhibitors can significantly reduce the pathogenicity of MDR *P. aeruginosa* strains; and thus can be an effective and successful strategy for treating antibiotic resistant traits.

**Supplementary Information:**

The online version contains supplementary material available at 10.1186/s12941-024-00684-5.

## Background

Antibiotic misuse, along with a reduction in the discovery of new antibiotics increased the number of multi-drug-resistant pathogens (MDRP) which are responsible for 700,000 deaths per year in the world. This issue will be more complicated by the year 2050 since the deaths in the world due to MDRP will be higher than the death rate in cancer patients. Several public health organizations have already declared that human beings will face the “disastrous consequences” of the antibiotic-resistance era that will lead to the devastation of human civilization [1].

Conventional antibiotics combat microbes by inhibiting protein synthesis, cell wall synthesis, and DNA replication that kill most pathogenic cells. This problem emerges when survived pathogen cells proliferate and develop a new community with an antibiotic resistance property. This classical treatment is replaced nowadays by an antibiotic-free strategy based on decreasing the virulence pathogenicity of MDRP without interfering with the growth cycle of a pathogen to threshold-inducing inactivation of the used drug [2]. The virulence of MDRP depends mainly on the communication among pathogen cells which is known as quorum sensing (QS). This system is a mechanism that helps the bacteria to regulate the gene expression of MDRP including antibiotic resistance, and modulation of host immune responses. Moreover, QS can occur either within the cells of the same pathogen species, among diverse species, or between microbial cells and the host immune system [3]. The QS machinery in pathogens produces diffusible signaling molecules called auto-inducers (AIs) or pheromones to communicate together. The most common auto-inducer molecules in Gram-positive bacteria belong to oligopeptides whereas n-acyl homoserine lactones (AHL) are predominant in Gram-negative bacteria [4].

*Pseudomonas aeruginosa* is a notorious Gram-negative, rod-shaped, non-fermentative bacterium that belongs to a γ-subdivision of the Proteobacteria. It is also considered a member of ESKAPE pathogens (*Enterococcus faecium*, *Staphylococcus aureus*, *Klebsiella pneumoniae*, *Acinetobacter baumannii*, *P. aeruginosa*, and *Enterobacter *sp.) that exhibits profound versatility in its virulence arsenal [5]. This opportunistic pathogen infects a wide range of hosts, including plants and animals as well as man. This human pathogen causes a wide array of life-threatening infections in the respiratory tract, primarily pneumonia, and chronic bronchitis in individuals suffering from cystic fibrosis and acquired immunodeficiency syndrome including cancer, organ transplantation, and AIDS patients. It also plays a role in developing severe eye infections including conjunctivitis, endophthalmitis, keratitis, dacryocystitis, and corneal ulceration with permanent vision loss [6]. This wide infectious diversity is associated with its ability to easily colonize epithelial surfaces, weaken host defenses, induce systemic toxicity, and consequently elevate morbidity and mortality rates (may reach 60%) in hospital-acquired infections. In addition, it can also colonize heart valves, catheters, or dental implants [7].

Unlike other MDRPs, *P. aeruginosa* can survive in any biological system exposed to high concentrations of antibiotics. It may also disturb the host microbiome which leads to increase in its virulence efficiency. The antibiotic resistance in *P. aeruginosa* is primarily mediated through two mechanisms; extrinsic or acquired resistance which is attained by mutational changes at the genetic level via horizontal gene transfer of β-lactamases or aminoglycosides modifying enzymes and adaptive resistance where transient alterations in gene expressions in response to environmental stimuli by restricting cell wall uptake, increasing efflux pump, and finally inactivating the inflexed drug [8]. The latter mechanism is easily reversed when the external stimuli are removed whereas the former one is permanent and emerges MDR *P. aeruginosa* strains that are extremely difficult to eradicate even with the third and the fourth generations of antibiotics [9]. The virulence of *P. aeruginosa* is correlated with pigment and rhamnolipids production, motility behavior, proteolytic enzymes, and biofilm formation and is stringently regulated by the QS system. This system plays a pivotal role in every stage of pathogenicity from the beginning of host colonization to invasion, dissemination, immune evasion, and finally drug resistance [6].

Therefore, many compounds either synthetic or with a natural origin that have structural similarities to autoinducer molecules can act as quorum-sensing inhibitors (QSI) and are used to inhibit or block the QS channels in *P. aeruginosa*. Halogenated furanones isolated from Australian red marine algae *Delisea pulchra* was considered as the first chemical class with a natural origin that plays a significant role in quorum sensing quenching [10]. Similarly, many approved drugs such as aspirin, niclosamide, salicylic acid, and sodium ascorbate, are efficient QSI at the transcriptional and expression of gene levels that lead to a significant reduction in virulence factors production [11]. Alternatively, gamma radiation as electromagnetic waves has a high penetration efficiency that removes electrons from atoms and molecules of any matter. Regarding biological systems, gamma irradiation causes deleterious effects due to damaging cell components especially proteins and nucleic acids by emitting free radicals (ROS) directly or indirectly, thereby impeding metabolic activity and causing cell death [12]. Hence, the present study aims to isolate and identify new compounds with QSI properties and investigate their impact as well as gamma irradiation on the virulence of MDR *P. aeruginosa* at the gene level.

## Material and methods

### Microorganisms and antibiotics

*Pseudomonas aeruginosa* U3 strain that was previously isolated from urinary tract infection [13] was kindly donated by Dr/Ahmed Omara, Microbiology Department, NCRRT, EAEA. Three pathogenic *P. aeruginosa* isolates out of forty-one pathogens were collected from the clinical microbiology laboratory of Demerdash General Hospital, Cairo, Egypt in 2019. Bacterial strains are identified by 16S rRNA gene sequencing as *P. aeruginosa* NCR-RT1, *P. aeruginosa* NCR-RT2, and *P. aeruginosa* NCR-RT3 and their nucleotide sequences were accessed in the GenBank as OQ271449, OQ271757, and OQ271760. The halophilic strains, *Halomonas cupida* Halo-Rt1, and *H. almeriensis* Halo-Rt5 were isolated from aquatic plants in saline ecosystems and *H. elongate* Halo-Rt2, *Sediminibacillus terrae* Halo-Rt4 were selected from Pharaoh’s spring where the former was a sandy soil origin and the latter was water aquatic origin. Moreover, *Vigibacillus natechei* Halo-Rt3 was isolated from seawater in Sharm El-Sheikh, South Saini governorate. The nucleotide sequences of selected halophiles were also accessed in the GenBank as follows, OQ271764, OQ272105, OQ271763, OQ271766, and OQ271765; respectively.

The most common antibiotics used in the antimicrobial susceptibility investigations for clinical cases were purchased from Bioanalyse Company; Amoxicillin-clavulanic acid (30 µg), Cefepime (30 µg), Meropenem (30 µg), Trimoxazole (25 µg), Gentamycin (10µg), Ceftazidime (30 µg), Tobramycin (10 µg), Ciprofloxacin (5 µg), and Amikacin (30 µg). The antimicrobial susceptibility was determined against the selected *P. aeruginosa* strains to select multi-drug resistant strains (MDR) using disc diffusion assay according to Clinical and Laboratory Standards Institute (CLSI) guidelines [14].

### Estimation of the virulence features in the selected MDR *P. aeruginosa* strains

In the present study, the virulence features of the selected MDR *P. aeruginosa* strains were studied under the stress of the sub-lethal dose of gamma irradiation (1kGy) or the sub-inhibitory concentrations of bioactive compounds recovered from halophilic bacterial strains in comparison to control (Untreated) where each MDR pathogen was irradiated with 1 kGy or was grown in the presence of sub-minimum inhibitory concentration of halophilic metabolites before estimating the virulence factors individually. Biofilm formation by the selected MDR *P. aeruginosa* strains was investigated by staining the formed biofilm with crystal violet (0.1%) for 10 min. after removing the planktonic cells. The absorbance of stained biofilm eluted with glacial acetic acid (30%) was measured at 550 nm using an ELIZA reader [15]. The rhamnolipid productivity of selected MDR pathogens was estimated after the formation of rhamnolipid–methylene blue complex in a non-polar solvent (chloroform) that was easily quantified using UV–vis spectrophotometer at 638 nm [16]. For estimating pyocyanin production, cultures of MDR pathogens (either irradiated or treated with halophilic metabolites) were grown in a glycerol-supplemented nutrient broth medium at 37 ℃ for 24 h under static conditions. Pyocyanin pigment was recovered using chloroform from the supernatant-free cells (pH 2) and its absorbance was measured at 520 nm and quantified as previously described [13]. Pyoverdine was quantified by measuring its fluorescence emission at an excitation wavelength (405nm) and an emission wavelength (465nm) using a multi-well fluorescence plate reader after diluting cell-free supernatants to ten folds by Tris–HCl buffer at pH 7.4 [17]. Proteolytic activity was determined using the skimmed milk assay where the clearance degree of skimmed milk was measured at 600 nm using a T60 UV–Vis spectrophotometer (China) and A lower degree of clearance is an indication of lower proteolytic activity [18]. The hemolysin assay was evaluated using fresh sheep red blood cells (2%). Released hemoglobin was measured at 540 nm in comparison with both negative control (erythrocytes incubated in LB broth) and positive control (erythrocytes lysed completely with 0.1% SDS). The hemolysis percentage was determined from the previously mentioned formula [19].

### Inhibiting quorum sensing systems in the selected MDR *P. aeruginosa* strains

Halophilic bacteria were grown independently in a balanced salt broth medium supplemented with 10% NaCl on a rotary shaker (125 rpm) for 8 days at 37 ℃. The bioactive compounds present in the cell-free filtrate were recovered using ethyl acetate. The solvent layers after segregation were concentrated by evaporating to dryness at 60 ℃, partially purified, weighed, and stored at 4 ℃ until analysis [20, 21]. Two-fold dilution of partially-purified halo-bacterial metabolites was adjusted for descending concentrations (500, 250, 125, 62.5, 31.25, 15.6, and 7.8 mg/ml) in the wells of microtiter plates. Then, every well received 100 µl of different *P. aeruginosa* cultures, 10µl of the resazurin indicator (0.062% w/v) and well mixed. Inoculated microplates were incubated overnight at 37 ℃, and the MIC value was determined by observing the lowest concentration of halo-bacterial metabolites that inhibited the growth of the selected MDR *P. aeruginosa* strains*.* The results of the minimum inhibitory concentration (MIC) of recovered bioactive compounds for each bacterial strain were summarized in Additional file 1: Table S1. Then, the efficiency of recovered bioactive compounds for inhibiting quorum sensing channels in selected MDR *P. aeruginosa* strains was determined.

The cells of the selected *P. aeruginosa* strains were individually irradiated at the log-growth phase to determine the sub-lethal dose. The irradiation process was carried out using the Indian Gamma Unit (Co_60_) at the National Center for Radiation Research and Technology (NCRRT), Nasr City, Cairo, Egypt; the dose rate was 1.57 kGy/h.

### Genetic detection of AHLs-dependent quorum-sensing genes

The genomic DNA of the selected *P. aeruginosa* strains was extracted using a commercial kit K0691 and purified using a commercial kit K0721 (GeneJet Genomic DNA Purification Kit, Thermo Fisher Scientific, USA). Two different sets of oligonucleotide primers (one set for intact gene amplicons and the second for internal fragment amplicons) corresponding to different regions in lasI, lasR, rhlI, and rhlR genes responsible for AHLs-dependent quorum-sensing were designed and synthesized by Invitrogen (Thermo Fisher Scientific). Another rhIR gene was also designed by the primer 3 plus program and symbolized as (REH). In addition, the primers of housekeeping control genes (rpoD, and ampC genes) were designed and amplified as the target QS genes. All primers used in the present study were tabulated in Additional file 1: Table S2. The reaction mixture of PCR was performed in a total volume of 25 µl containing 10 pmol of forward and reverse primers. The amplification reaction for genes under investigation was performed in PCR (Lapcycler Basic and Labcycler Gradient, SensoQuest Biomedical Electronic, German). The thermal cycle parameters were as follows: initial denaturation at 95 ℃ for 5 min, followed by 30 cycles for 35 s at 95 ℃, 53 ℃ for 40 s, 72 ℃ for 1 min, and finally an extension cycle at 72 ℃ for 10 min. Synthesized DNA fragments were detected on 1% agarose gels by ethidium bromide staining [22]. Gene ruler plus 100 bp DNA was used as the standard. Bands were screened using a UV-imaging system (UV transilluminator, Wealtec, USA) and analyzed with the integrated software [23].

### Quantitative RT-PCR of QS genes

RNA of the selected *P. aeruginosa* strains either treated with halo-bacterial metabolites or exposed to 1kGy in comparison to control (untreated) was extracted using GeneJET RNA Purification Kit K0731 (Thermo Scientific, USA) following the manufacturer’s instructions, and the purified RNA was stored at − 80 ℃ until use. To avoid any genomic DNA contaminant, a double DNA digestion treatment was performed [24]. The purified RNA was assessed by observing the ratio of its absorbance at 260 and 280 nm using a T60 UV–vis Nanodrop Spectrophotometer (China); pure RNA has a ratio of around 2.1 [25]. cDNAs were synthesized via reverse transcription using 10 ng of mRNA according to the instructions of cDNA synthesis kit PN 4374966 (High Capacity cDNA Transcription Kits, Applied Biosystems by Thermo Fisher Scientific, USA). The thermal program started at 25 ℃ for 10 min. (Annealing step), then 120 min. at 37 ℃ (cDNA synthesis step) followed by 5 min. at 85 ℃ (Denaturation step) and holding at 4 ℃. The cDNA was stored at – 80 ℃ until use. PCR amplification for all cDNAs was performed using a real-time fluorescence thermal cycler (Rotor-Gene 2000, Corbett Ltd., Australia) with a heated lid (105 ℃) based on PCR programs. The PCR-heat program was started with an initial denaturation step at 95 ℃ for 15 s, followed by an annealing step that lasted for 20 s at 55 ℃, and the extension cycle (20 s) was repeated for 40 cycles at 72 ℃. Each cDNA fragment was amplified in duplicate for each sample. The expression of housekeeping control genes (rpoD and ampC) was normalized between samples in each set thus, the relative amount of QS gene expression could be determined from the standard curves. The comparative threshold cycle (2^ΔΔCt^) method was used to calculate relative gene expression [26].

### Characterization of halo-bacterial metabolites using liquid chromatography–mass spectroscopy (LC/MS) analysis

The chemical composition of halo-bacterial metabolites was determined using liquid chromatography–ionization–tandem mass spectrometry (LC–ESI–MS/MS) with an Exion LC system for separation and SCIEX Triple Quad 5500 + MS/MS system equipped with electrospray ionization (ESI) for detection. The separation was performed with an Ascentis® Express 90 Å C18 Column (2.1 × 150 mm, 2.7 µm). The mobile phases consisted of two eluents; A: 5 mM ammonium formate (pH 3) and B: acetonitrile (HPLC grade). The elution of the mobile phase was as follows; 5% B eluent for 1 min, 5–100% B eluent for 19 min, 100% B eluent from 5 min. followed by 5% A eluent for 5 min. The flow rate was 0.3 ml/min.

For MS/MS analysis, positive ionization mode was applied with a scan (EMS-IDA-EPI) from 100 to 1000 Da for MS1 with the following parameters: curtain gas: 25 psi; Ion Spray voltage: 5500, and source temperature: 500 °C.

## Results

### The virulence features in the MDR *Pseudomonas aeruginosa* strains after treatment with halo-bacterial metabolites or gamma irradiation

In a preliminary experiment, four clinical isolates belonging to *Pseudomonas aeruginosa* species showed antibiotic resistance to seven antibiotics; amoxicillin-clavulanic acid, cefepime, trimoxazole, gentamycin, ceftazidime, tobramycin, and ciprofloxacin. Alternatively, selected halophilic bacterial strains (*Halomonas cupida* Halo-Rt1, *H. elongate* Halo-Rt2, *Vigibacillus natechei* Halo-Rt3, *Sediminibacillus terrae* Halo-Rt4, and *H. almeriensis* Halo-Rt5) previously revealed antagonistic activity against two MDR *P. aeruginosa* out of five strains (Unpublished data).

The impact of partially-purified metabolites recovered from selected halophiles at sub-inhibitory concentration and gamma irradiation (at the sub-lethal dose) on the virulence features of *P. aeruginosa* including biofilm formation, rhamnolipid, pyocyanin, pyoverdin production, protease, and hemolysin activity was studied and summarized in Table 1. Gamma rays completely inhibited pyocyanin and pyoverdin pigment production as well as hemolysin activity for all *P. aeruginosa* strains under investigation. For most treatments, 1 kGy decreased both proteolytic activity and biofilm formation by 70% or more. The positive effect of gamma rays on rhamnolipid production was observed after exposing *P. aeruginosa* NCR-RT2 and *P. aeruginosa* NCR-RT3 to the sub-lethal dose (The increase was nearly 20% and 200%; respectively). Contrary, partially purified metabolites recovered from *H. elongate* Halo-Rt2, *S. terrae* Halo-Rt4, and *H. almeriensis* Halo-Rt5 decreased the rhamnolipid production by *P. aeruginosa* U3, *P. aeruginosa* NCR-RT2, and *P. aeruginosa* NCR-RT3; respectively by 100%. The proteolytic activity of the latter *P. aeruginosa* strains declined to less than 10% after treatment with metabolites recovered from *H. cupida* Halo-Rt1, *H. elongate* Halo-Rt2, and *H. almeriensis* Halo-Rt5; on the same order. Interestingly, the metabolites of *H. elongate* Halo-Rt2 decreased the pyocyanin production in *P. aeruginosa* NCR-RT2 by 100% whereas the metabolites of *H. cupida* Halo-Rt1 enhanced the same pigment production in *P. aeruginosa* NCR-RT3 by 50%. The decrease in other treatments was nearly 40% or more. Regarding biofilm formation, the most efficient treatment was accomplished by metabolites recovered from *S. terrae* Halo-Rt4 since the decrease in the formation reached 70% for *P. aeruginosa* NCR-RT2. The pyoverdin pigment production in *P. aeruginosa* NCR-RT3 declined by half or more when treated with metabolites of *H. cupida* Halo-Rt1, and *H. almeriensis* Halo-Rt5. The decrease in hemolysin activity of the selected *P. aeruginosa* strains was in the range between 50 and 75% after exposure to sub-inhibitory concentrations of partially-purified halophiles’ metabolites in comparison to control (Untreated).

**Table 1:** Evaluating the virulence features in the selected MDR *Pseudomonas aeruginosa* strains treated with metabolites of halophilic bacteria or gamma irradiation

Selected *Pseudomonas aeruginosa *strains	Treatment	The change in the yield of virulence factors in *P. aeruginosa* strains after treatment compared to control (100%)					
		Biofilm (%)	Rhamnolipids (%)	Pyocyanin (%)	Pyoverdin (%)	Protease (%)	Hemolysins (%)
*P. aeruginosa* U3	Gamma irradiation (1 kGy)	63.00 ± 2.83	47.24 ± 0.99	–	–	27.00 ± 1.41	–
	*Halomonas cupida* (Halo-Rt1)	81.00 ± 1.41	56.08 ± 5.66	14.92 ± 0.06	56.70 ± 1.24	3.00 ± 1.41	54.00 ± 0.01
	*Halomonas elongate* (Halo-Rt2)	60.00 ± 0.02	–	37.97 ± 0.03	61.00 ± 0.91	32.1.41	61.00 ± 0.92
	*Vigibacillus natechei* (Halo-Rt3)	ND	ND	ND	ND	ND	ND
	*Sediminibacillus terrae* (Halo-Rt4)	ND	ND	ND	ND	ND	ND
	*Halomonas almeriensis* (Halo-Rt5)	ND	ND	ND	ND	ND	ND
*P. aeruginosa* NCR-RT1	Gamma irradiation (1 kGy)	26.00 ± 0.01	86.57 ± 0.28	–	–	25.00 ± 0.01	–
	*Halomonas cupida* (Halo-Rt1)	79.00 ± 5.66	32.06 ± 0.56	42.65 ± 0.08	86.00 ± 1.41	86.00 ± 0.01	50.00 ± 2.12
	*Halomonas elongate* (Halo-Rt2)	ND	ND	ND	ND	ND	ND
	*Vigibacillus natechei* (Halo-Rt3)	71.00 ± 0.02	32.06 ± 0.14	64.70 ± 0.04	90.00 ± 1.41	86.00 ± 1.41	43.00 ± 1.70
	*Sediminibacillus terrae* (Halo-Rt4)	ND	ND	ND	ND	ND	ND
	*Halomonas almeriensis* (Halo-Rt5)	ND	ND	ND	ND	ND	ND
*P. aeruginosa* NCR-RT2	Gamma irradiation (1 kGy)	35.00 ± 1.41	118.15 ± 0.01	–	–	6.00 ± 1.41	–
	*Halomonas cupida* (Halo-Rt1)	ND	ND	ND	ND	ND	ND
	*Halomonas elongate* (Halo-Rt2)	61.00 ± 1.41	41.38 ± 0.01	–	73.00 ± 1.4	9.00 ± 2.83	–
	*Vigibacillus natechei* (Halo-Rt3)	ND	ND	ND	ND	ND	ND
	*Sediminibacillus terrae* (Halo-Rt4)	30.00 ± 0.20	–	18.90 ± 0.04	83.00 ± 2.83	11.00 ± 0.01	75.00 ± 0.02
	*Halomonas almeriensis* (Halo-Rt5)	ND	ND	ND	ND	ND	ND
*P. aeruginosa* NCR-RT3	Gamma irradiation (1 kGy)	34.00 ± 0.01	206.40 ± 1.3	–	–	28.00 ± 0.01	–
	*Halomonas cupida* (Halo-Rt1)	81.00 ± 0.71	53.52 ± 0.01	152.50 ± 0.03	50.00 ± 0.01	2.00 ± 0.01	65.00 ± 2.12
	*Halomonas elongate* (Halo-Rt2)	ND	ND	ND	ND	ND	ND
	*Vigibacillus natechei* (Halo-Rt3)	ND	ND	ND	ND	ND	ND
	*Sediminibacillus terrae* (Halo-Rt4)	ND	ND	ND	ND	ND	ND
	*Halomonas almeriensis* (Halo-Rt5)	64.00 ± 0.01	–	33.35 ± 0.02	40.00 ± 1.41	7.00 ± 0.01	58.00 ± 0.01

–: It means 0%; ND: it means not detected in this set of experiments because of negative results in the screening experiment

### Genotyping of AHLs-dependent quorum-sensing systems in the MDR *P. aeruginosa* strains after treatment with halo-bacterial metabolites or gamma irradiation

**Fig. 1:**
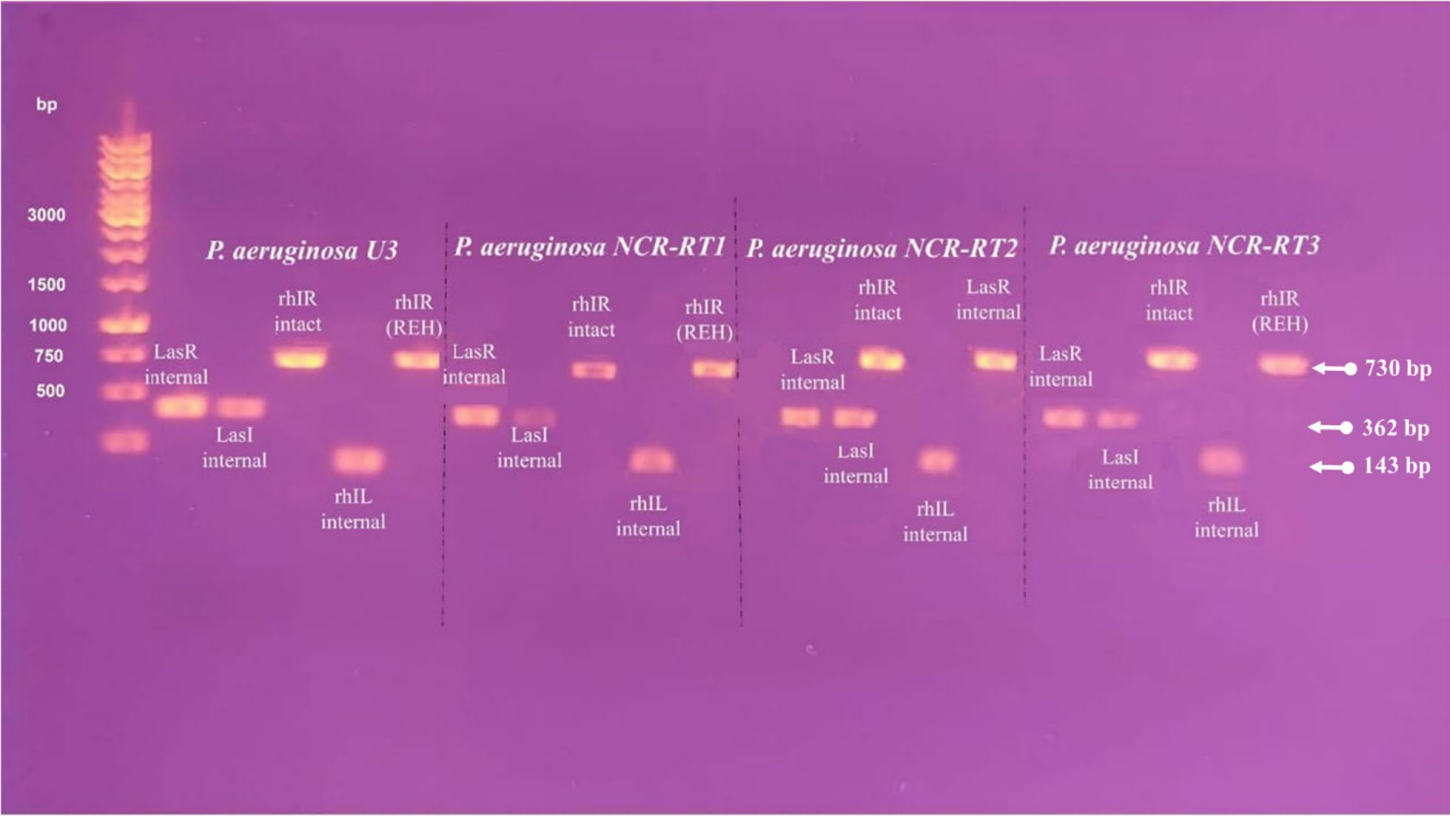
Agarose gel electrophoresis for PCR products showing las (LasI/LasR), and rhl (rhlI/rhlR) of AHLs-QS system

Figure 1 shows the presence of the main QS systems; las (LasI/LasR), and rhl (rhlI/rhlR), which are regulated by auto-inducers belonging to n-acyl-homoserine lactones (AHLs) in four MDR *P. aeruginosa* strains under investigation (The full uncropped agarose gel was shown in supplementary material as an image, Additional file 1: Fig. S3). Not all intact and internal primers could be polymerized into the desired QS genes as observed in Fig. 1. The amplified PCR products of rhlI (internal) had approximately 143 bp whereas both of lasI (internal) and LasR (internal) were amplified with 362pb. The amplified rhlR (intact) was 730 bp as same as the rhlR (REH).

**Fig. 2:**
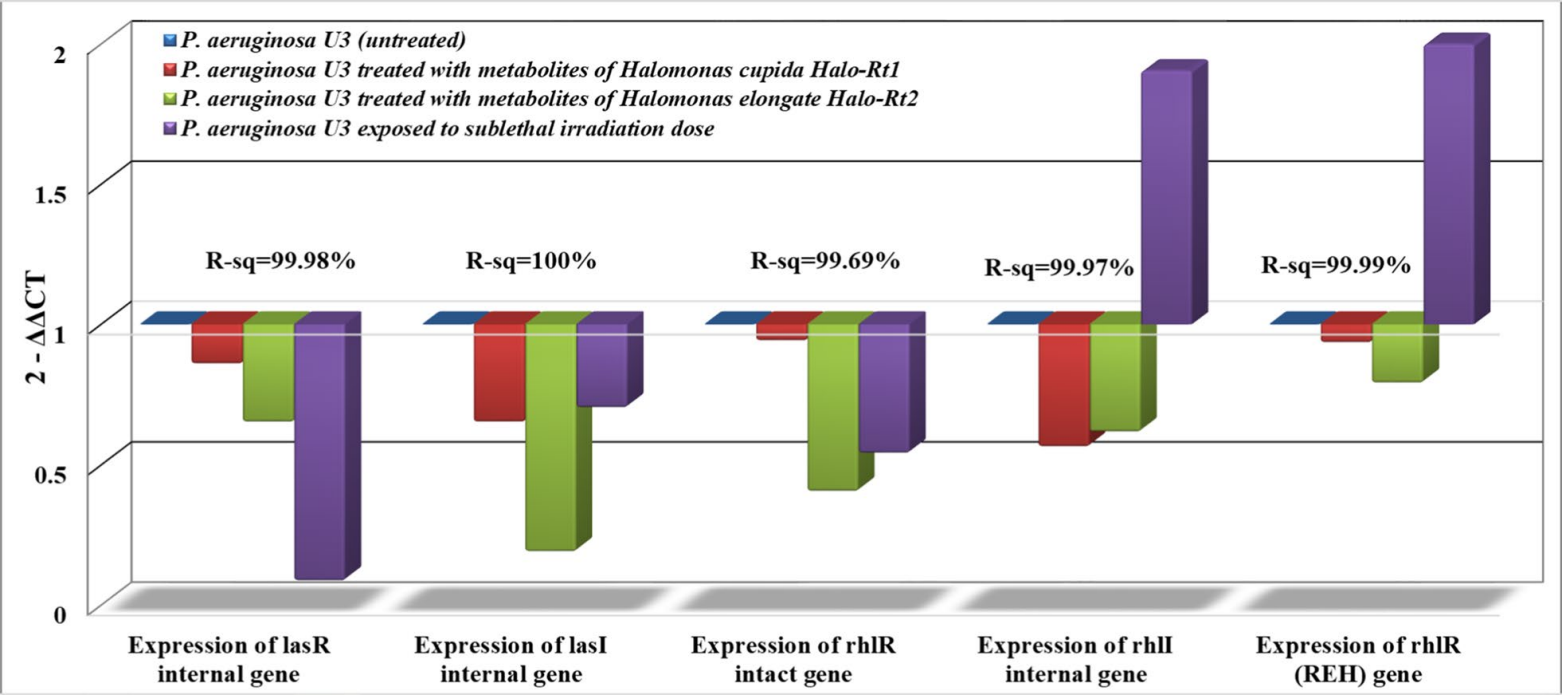
Expression of AHLs-dependent QS genes in *P. aeruginosa U3* treated with metabolites of halophiles or γ-irradiation in comparison with the untreated group

**Fig. 3:**
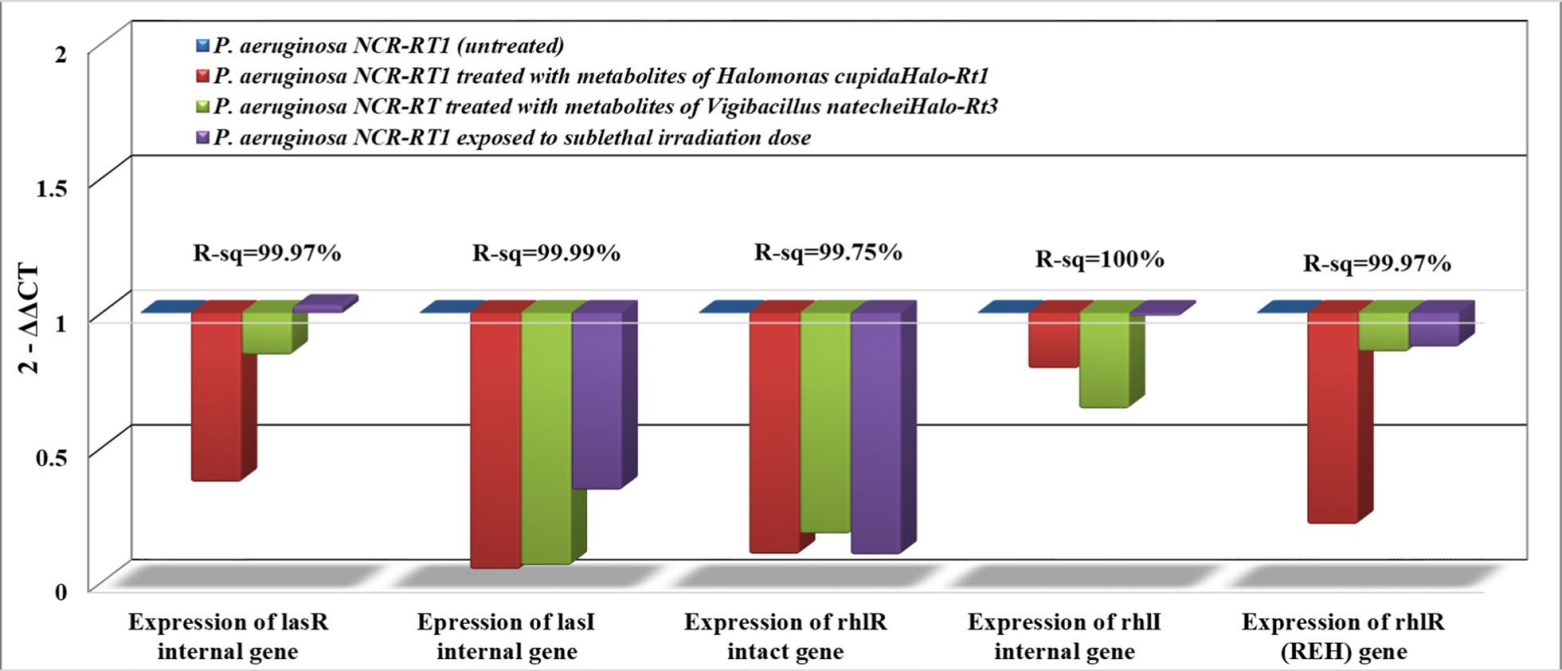
Expression of AHLs-dependent QS genes in *P. aeruginosa* NCR-RT1 treated with metabolites of halophiles or γ-irradiation in comparison with the untreated group

**Fig. 4:**
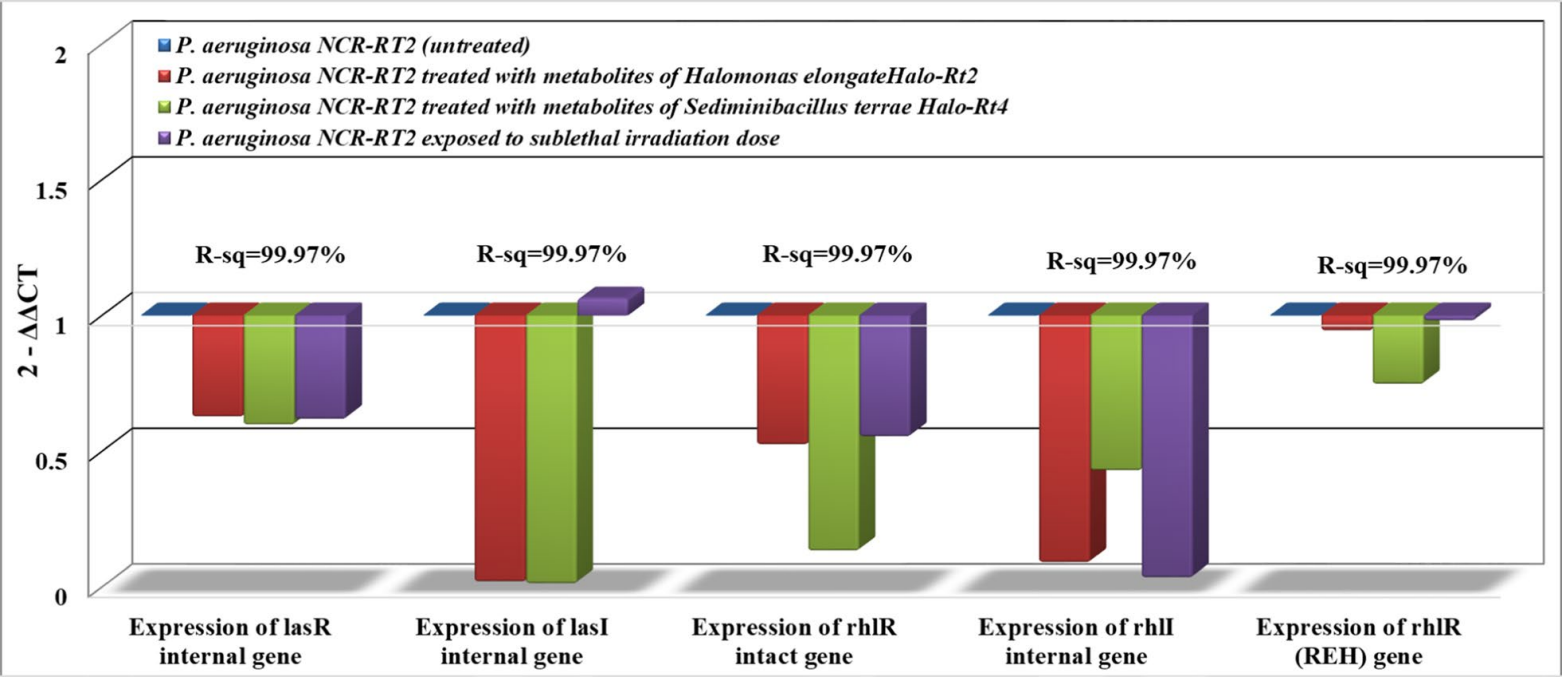
Expression of AHLs-dependent QS genes in *P. aeruginosa* NCR-RT2 treated with metabolites of halophiles or γ-irradiation in comparison with the untreated group

**Fig. 5:**
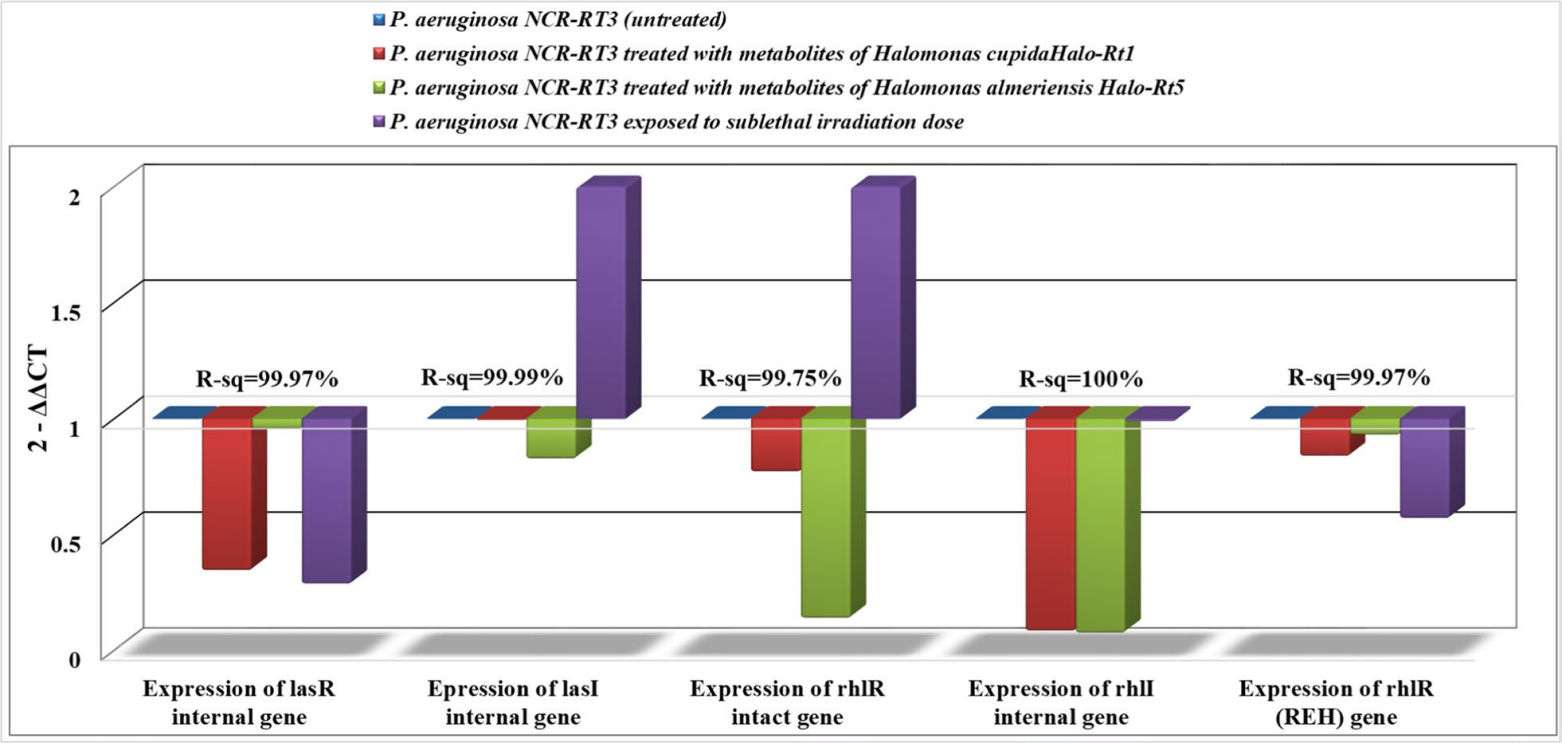
Expression of AHLs-dependent QS genes in *P. aeruginosa* NCR-RT3 treated with metabolites of halophiles or γ-irradiation in comparison with the untreated group

The impact of stressed variables (Gamma irradiation and Halo-bacterial metabolites) on the expression of AHLs -dependent QS genes (LasR internal, LasI internal, rhlR intact, and rhlI internal) in comparison to rhlR (REH) were determined by quantitative real-time PCR and the results are shown in the Figs. 2, 3, 4, and 5. The main result in all treatment groups under investigation was a decrease in the expression level in comparison with the control (untreated group).

Figure 2 reveals the negative impact of halophilic metabolites recovered from *H. cupida* Halo-Rt1, and *H. elongate* Halo-Rt2 on the expression of AHLs-dependent QS genes in *P. aeruginosa* U3. Results showed the superiority of metabolites recovered from *H. elongate* Halo-Rt2 over those extracted from *H. cupida* Halo-Rt1 in the expression of all genes under investigation; the expression of LasI gene was decreased by nearly 80% (0.1881^2−ΔΔCT^ ± 0.007^c^) in comparison to the control (1^2−ΔΔCT^ ± 0.001^a^). The same pattern was observed for the impact of the sub-lethal dose of irradiation on three QS genes including LasR internal, LasI internal, and rhlR intact whereas the irradiation increased the expression of the rhlI internal, and rhlR (REH) genes by nearly two and three folds respectively. A ten-fold reduction in the expression of the LasR internal gene was achieved by irradiating *P. aeruginosa U3* with 1 kGy where the expression decreased from 1^2−ΔΔCT^ ± 0.001^a^ with the sub-lethal dose of irradiation to 0.0847^2−ΔΔCT^ ± 0.004^d^.

The expression of the AHLs-dependent QS genes in *P. aeruginosa* NCR-RT1 following individual treatment with MIC of halo-bacterial metabolites and the sub-lethal dose of irradiation was shown in Fig. 3. The expression of the LasI internal gene was decreased to nearly twenty folds; 0.0451^2−ΔΔCT^ ± 0.004^b^ and 0.0595^2−ΔΔCT^ ± 0.007^c^ in comparison with the control (1^2−ΔΔCT^ ± 0.001^a^) due to the metabolites of *H. cupida* Halo-Rt1 and *V. natechei* Halo-Rt3; respectively. This was followed by a ten-fold decrease in the rhlR intact gene expression (0.1015^−ΔΔCT^ ± 0.001^a^, and 0.1767^2−ΔΔCT^ ± 0.001^b^) after treating cells of *P. aeruginosa* NCR-RT1 with the same halo-bacterial metabolites; respectively. Similar to previous results observed in *P. aeruginosa U3*, gamma irradiation at the sub-lethal dose negatively affected the expression of all AHLs-dependent QS genes in *P. aeruginosa* NCR-RT1 except LasR internal gene (1.0281^2−ΔΔCT^ ± 0.001^c^). The least expressed gene was the rhlR intact gene (0.0994^2−ΔΔCT^ ± 0.001^c^), therefore the decrease was more than tenfold.

Results in Fig. 4 show a decrease in the expression of the lasR internal gene upon treating *P. aeruginosa* NCR-RT2 cells with irradiation or halo-bacterial metabolites (recovered from *H. elongate Halo-Rt2*, *S. terrae Halo-Rt4*) by nearly 40% in comparison to untreated cells (control). A greater reduction in the expression of lasI internal gene (ninety folds or more) was achieved after exposing *P. aeruginosa* NCR-RT2 cells to the previously mentioned metabolites (0.0169^2−ΔΔCT^ ± 0.0002^b^ and 0.0103^2−ΔΔCT^ ± 0.0002^c^; on the same order). Irradiation succeeded in decreasing the expression of the rhlI gene in *P. aeruginosa* NCR-RT2 cells by more than thirty folds; 0.0319 ± 0.001^d^ in comparison to 1.0 ± 0.001^a^ of the control. Alternatively, the expression of the lasR internal gene was slightly changed by irradiation (1 kGy). The rhIR (REH) gene showed the least expression capacity (> 25%) in comparison to other Qs genes under investigation.

The expression of selected AHLs-dependent QS genes in *P. aeruginosa* NCR-RT3 treated with metabolites of *H. cupida* Halo-Rt1, and *H. almeriensis* (sub-inhibitory concentrations) as well as irradiation (1 kGy) in comparison with the untreated group was illustrated in Fig. 5. The expression of rhlI internal gene was reduced by more than ten folds after treating with halophilic metabolites recovered from *H. cupida* Halo-Rt1 (0.0807^2−ΔΔCT^ ± 0.0001^b^) and *H. almeriensis* Halo-Rt5 (0.0722^2−ΔΔCT^ ± 0.001^c^). Similarly, the expression of the rhlR intact gene was downregulated to 0.1358^2−ΔΔCT^ ± 0.009^c^ when *P. aeruginosa* NCR-RT3 cells were treated with metabolites recovered from *H. almeriensis Halo-Rt*5 whereas the metabolites of *H. cupida Halo-Rt1* declined the expression of the lasR internal gene to nearly one-third (0.3415^2−ΔΔCT^ ± 0.013^b^). Contrary, irradiating *P. aeruginosa* NCR-RT3 cells with 1 kGy increased the expression of the lasI internal gene and rhlR intact gene by two folds since they recorded 2.0849^2−ΔΔCT^ ± 0.002^c^ and 2.1584^2−ΔΔCT^ ± 0.082^d^, respectively. On the other hand, there was no obvious difference in rhlI internal gene expression after exposure to irradiation compared to the untreated control.

Using LC–ESI–MS/MS, a variety of chemical compounds with quorum quenching capabilities were found in the partially-purified metabolites recovered from *H. cupida* (Halo-Rt1) including glabrol, 5,8-dimethoxyquinoline-2-carbaldehyde, linoleoyl ethanolamide, agelasine, penigequinolones derivatives, berberine, tetracosanoic acid, and liquidambaric lactone. Regarding *H. elongate* (Halo-Rt2), four QSI were identified, they are phloretin, lycoctonine, fucoxanthin, and crassicauline A in the latter one (Table 2).

**Table 2:** Identification of quorum sensing inhibitors in bioactive metabolites recovered from *Halomonas cupida* (Halo-Rt1) and *Halomonas elongate* (Halo-Rt2)

Halophilic bacterial strains	Prospected quorum-sensing inhibitors (QSI)	RT (min.)	Molecular weight	Molecular formula	Mechanism of QSI
*Halomonas cupida*	Glabrol	5.92	415.2	C_25_H_28_O_4_	No previous reports
	5,8-dimethoxyquinoline-2-carbaldehyde	8.56	218.04	C_12_H_11_NO_3_	No previous reports
	Linoleoyl ethanolamide	13.58	324.36	C_20_H_37_NO	No previous reports
	Agelasine	19.59	458.28	C_26_H_40_N_5_	Inhibiting biofilm formation [51]*
	Penigequinolones derivatives	20.44	468.24	C_16_H_20_N_2_O_6_	No previous reports
	Berberine	22.58	336.12	C_20_H_18_NO	Disrupting motility, biofilms, pyocyanin, and efflux pumps [53]*
	Tetracosanoic acid	23.20	391.32	C_24_H_48_O	No previous reports
	Liquidambaric lactone	24.88	469.32	C_30_H_44_O_4_	No previous reports
*Halomonas elongate*	Phloretin	8.89	459.18	C_15_H_14_O	Decreasing swarming and swimming motility [55]*
	Lycoctonine	17.73	468.24	C_25_H_41_NO_7_	No previous reports
	Fucoxanthin	19.14	681.24	C_42_H_58_O_6_	Decreasing cell adhesion through the blocking of quorum sensing [56]*
	Crassicauline	20.15	644.28	C_35_H_49_NO_10_	No previous reports

^*^They are mentioned in the references section

## Discussion

Quorum sensing is responsible for regulating about 10% of *Pseudomonas aeruginosa* genes and about 20% of its proteomic yield. The quorum-sensing system depends on auto-inducers (signaling molecules) produced by the bacterial population. At low cell density, these signaling molecules diffuse away without causing any detectable harm until the population extends a certain limit. With a high cell density, these molecular signals are easily detected by certain receptors on the cell membrane or in the cytoplasm resulting in a cumulative production and expression of pathogenicity genes [27]. *P. aeruginosa* possesses four QS systems (las, rhl, Iqs, and Pqs), the main QS channels; las (lasI/lasR), and rhl (rhlI/rhlR), which are regulated by auto-inducers belong to N-acyl-homoserine lactones (AHLs). The las system comprises lasI, which is a Lux1-type synthase responsible for the synthesis of autoinducer; n-(3-oxododecanoyl)-l-homoserine lactone (3O-C12-HSL, odDHL). This autoinducer is responsible for the bacterial efflux pump mechanism and can bind to the transcriptional activator lasR. The lasR-3-oxo-C12-HSL complex activates the transcription of rhlR, rhlI, and lasI resulting in a positive feedback loop [6]. The multimeric forms of lasR induce the target genes of biofilm formation, hemolysin, proteases, elastases, and exotoxin-A production, and regulate their transcription. Additionally, lasR also induces the expression of a transcription repressor of lasI (RsaL). It acts as a negative feedback loop that contradicts the positive feedback loop thus both of them balance the levels of signal molecules of QS [28]. In rhl system, rhlI is also Lux1-type synthase that produces the autoinducer; AHL n-butyryl-l-homoserine lactone (C4-HSL, BHL). It mediates passive diffusion and can bind to rhlR, as the transcriptional regulator for several genes involved in pathogenicity including rhamnolipid, alkaline protease, elastase, cyanide, and pyocyanin production [29]. The rhlR-C4-HSL complex easily dimerizes and activates rhlI, therefore, it could be considered a second positive feedback loop in the QS system. All the quorum-sensing systems are closely related to each other and do not exist independently. They form together an intricate hierarchical quorum-sensing circuitry in which las is at the top of the QS hierarchy. This explains the decline in the rhlI/R expression after decreasing the expression in lasI/R system as shown in Figs. 2, 3, 4. The las system positively regulates the expression of both rhlR and rhlI. Thus, lasI/R controls the first wave of quorum-sensing–controlled gene expression followed by rhlI/R in a synchronized arrangement system [30].

The other QS system in *P. aeruginosa* is a non-AHL-mediated QS signaling (IQS & PQS); Iqs system employs 2-(2-hydroxyphenyl)-thiazole-4-carbaldehyde, which is supposed to regulate the Pqs system that is associated with the production of 2-heptyl-3-hydroxy-4-quinolone; a signaling molecule that transported by outer membrane vesicles [31]. It induces the expression of genes involved in the swimming motility and biofilm formation as well as the production of proteases, elastases, rhamnolipids, pyocyanin, and pyoverdine siderophores thus, facilitating immune evasion and destruction of the immune cells such as macrophages and neutrophils. Moreover, Pqs involved in the regulation of rhlI expression by influencing the C4-HSL output thereby cumulatively impact on the rhl system, in the meanwhile, the production and activity of Pqs are dependent on lasR and rhlR. The expression of the PqsR and Pqs operons is inhibited by rhlRC4-HSL, suggesting that the concentration ratio between 3-oxo-C12-HSL and C4-HSL plays a critical role in the dominance of the Pqs signaling system [6]. The four QS channels; las, rhl, Iqs, and Pqs are dependent on the transcriptional regulators; lasR, rhlR, IqsR, and PqsR, respectively which are known as multiple virulence factor regulator (MvfR) that initiate the expression of the virulence genes [32]. Although seven primers are used to qualify AHLs-dependent QS genes as mentioned in the materials and methods section, five only can be detected due to the presence of sufficient base pair mismatches in the region of the undetected primers that prevent their successful amplification into a gene product or the treatment of selected *P. aeruginosa* strains with irradiation or halo-bacterial metabolites may convert the QS structural genes into non-functional due to mutations within one or more points. In the following discussion, we will outline the relation between the quorum sensing system and the expression of main virulence factors.

Pyocyanin is a redox-active compound that gives *P. aeruginosa* colonies a blue-green shade and disturbs multiple host cell functions such as catalase activity, electron transport in mitochondria, metal-ion uptake, and apoptosis of phagocytic cells because of its easily penetrating efficiency in biological membranes [33]. The synthesis of pyocyanin is widely regulated by the QS system, especially the transcriptional activator of Pqs system; PqsR as well as the regulatory signals of the las and rhl systems [28]. The microbial production of pyocyanin is a complicated process in which two identical operons; phz1 and phz2 and two modifying enzymes, PhzM and PhzS are involved in a QS-driven dependent process [34]. A significant decrease in the activity of the phz2 promoter was observed for *P. aeruginosa* strain that was unable to produce either HHQ or Pqs [35]. It was reported that the increase in the susceptibility of *P. aeruginosa* to oxygen-free radicals is correlated with the inhibition of pyocyanin [36]. In the present study, a complete inhibition of pyocyanin was observed in all *P. aeruginosa* strains under the oxidative stress conditions induced by gamma rays. Gamma rays primarily induce ROS production by exciting endogenous photosensitizing compounds that occur naturally within microbial cells or releasing singlet oxygen and hydroxyl radicals from growing cultures, leading to the destruction of target cells and various cellular components including membranes, proteins, nucleic acids, and lipids. This storm of destruction leads to a decrease in the pathogenicity [37].

In addition, previous findings revealed that the regulator PqsR directly activates the expression of nearly 35 loci, including the operon pqsABCDE encoding the enzymes involved in the synthesis of non-AHL-mediated QS autoinducers and for the expression of PqsE protein which shares with the rhl system in regulating the synthesis of pyocyanin. Similarly, it was found that both ginkgolic and hydroginkgolic acids, the main bioactive compounds in *Pistacia lentiscus* could attenuate the virulence of *P. aeruginosa* by interfering with the production of 4-hydroxy-2-alkylquinolines molecules, thus disturbing Pqs channel and lowering the pyocyanin yield [38]. Thus, the two phz operons may be regulated by the hierarchal network of all QS systems in *P. aeruginosa*. i.e. the switching off for any QS system is compensated by activating another thus a complete blocking of all QS channels couldn’t be achieved. This could explain the hesitant effect of halo-bacterial metabolites on pyocyanin production by the selected *P. aeuroginosa* strains.

*P. aeruginosa* possesses two major siderophores; pyocheline and pyoverdine that are responsible for iron capturing from the host tissues to provide the pathogen with sufficient amounts of iron necessary for proliferation and survival where the affinity of the latter siderophore is higher than the former one [39]. Therefore the present study follows its productivity in the selected MDR *P. aeruginosa* strains under stress conditions. As shown in Table 1, gamma irradiation succeeded in quantitatively suppressing the pyoverdin production in a higher yield than another treatment based on bioactive metabolites. Three classes of pyoverdine were previously identified as PvdI, PvdII, and PvdIII, they can chelate the Fe (III) dissolved in the external medium. Synthesis of the siderophore pyoverdine is induced by the las system but requires rhlR-C4-AHL to full activation; this means the regulatory systems show considerable overlap between las and rhl systems for accomplishing virulence features.

The major classes of enzymes that induce the pathogenesis of *P. aeruginosa* are proteases and lipases. About 2.8% of the genome in *P. aeruginosa* is directed toward the expression of hallmark protease genes containing serine, cysteine, threonine, and aspartate. The regulation of protease depends mainly on the las and rhl systems [40]. Similar to the results of this study, it was found that the pathogenicity virulence of *Staphylococcus aureus* could be decreased especially α-hemolysin and protease activities upon exposing cells to laser light in the presence of methylene blue [41].

Pathogens protect their cells with an extracellular polymeric substance called biofilm that is composed of complex polysaccharides, lipids, and proteins. It provides pathogen cells with superior performance including physical protection against antibiotics and/or drugs, nutrient storage, desiccation tolerance, and adhesion to host cells. The biofilm layer reduces the membrane permeability and acts as a diffusion barrier due to the presence of constitutive and inducible efflux pumps, which leads to a reduction in the rate of antibiotic penetration and the accumulation of antibiotics to limit their toxicity until the resistance genes would be expressed [42]. The bacterial layers in the biofilm are heterogeneous where the inner layers are less active than the outer due to low access to oxygen thus the potency of antibiotics is more obvious in cells on the surface of the biofilm but the inner layers remain unaffected, which increases the virulence potency. QS system appears to be involved in all biofilm formation steps, such as microbial surface attachment, exopolysaccharide matrix production, and biofilm detachment or degradation [43]. In a previous study, spatial configuration revealed that the expression of lasI and rhlI sharply decreased with increasing biofilm height, or with increasing the number of cells at the substratum [44]. A bacterial mutant can’t produce the las signal molecule (3O-C12-HSL) and fails to form biofilms with three-dimensional thickness in comparison to the parent strain. In contrast, a rhlI *P. aeruginosa* mutant that closely resembled the parent strain couldn’t develop biofilms. This suggests that the las QS system, not the rhl QS system is critical for biofilm formation [45]. In the present study, results showed the efficiency of bioactive metabolites recovered from *Halomonas* spp as well as irradiation in the reduction of biofilm formation by the selected MDR *P. aeruginosa* strains (Table 1) and subsequently, the decrease in lasI expression Figs. 2 and 5. QS-deficient mutants of *P. aeruginosa* with lower expression efficiencies for lasR rhlR and lasI rhlI, exhibited distorted biofilm architecture with thin, underdeveloped structures and susceptibility to antibiotics. It was found that the increase in a key QS-signaling molecule; cyclic-di-GMP controls the biofilm formation in *P. aeruginosa* by inducing the expression of adhesions and matrix components [46]. Moreover, QS-mediated production of pyocyanin was a positive regulator for biofilm formation by promoting the release of extracellular DNA [28]. The irradiated *P. aeruginosa* strains in the present study showed a decrease in pyocyanin pigment production and biofilm formation where the decrease was more obvious in the former variable than in the latter one.

Rhamnolipids are composed of l-rhamnose and β-hydroxy fatty acid; it may be monorhamnolipid, or dirhamnolipid. They primarily maintain the cell surface hydrophobicity and participate in the ingestion of insoluble substrates especially hydrocarbons to facilitate their utilization as carbon sources [47]. Rhamnolipids are the virulence molecules under the control of *P. aeruginosa* quorum sensing and share in the pathogenicity against host cells including lysis of macrophages, polymorphonuclear leukocytes, and evasion from the host immune system. It was reported the enhancement of rhamnolipids production under stress conditions including nutrient starvation, iron-limiting conditions, and phosphate starvation. Similarly, results in Table 1 showed an increase in rhamnolipids yield of *P. aeruginosa* NCR-RT3 and *P. aeruginosa* NCR-RT2 by 206.40 ± 1.3% and 118.15 ± 0.01%; respectively under the stress of gamma irradiation (1kGy). Under nutrient-limited conditions, the expression of las and rhl genes was predominant during the mid-log phase whereas PQS was released in the late growth-exponential phase [48]. Rhamnolipids induce the detachment of biofilm cells from the supporting surface and convert the bacterial layers into the planktonic state; this reverse relation was obvious in the phenotype virulence factors of most *P. aeruginosa* strains stressed by gamma irradiation in the present study. It has been previously reported that rhamnolipids production occurs in rhlAB operon and rhlC gene under the control of QS genes, particularly the transcriptional regulator rhlR, even under low levels of AHLs [49]. In the present study, the upregulation of rhlR after treating *P. aeruginosa* NCR-RT3 cells with bioactive metabolites recovered from *Vigibacillus natechei* (Halo-Rt3) and gamma irradiation; reached twenty folds or more. It was revealed that rhamnolipids production increased in a lasR-independent manner via inducing rhlR expression by sigma factor 54 (σ54). Alternatively, a previous study reported that rhamnolipids production is mildly activated by the las and pqs systems [6].

QSIs were defined as compounds that either inhibit or stimulate QS-regulated gene expression, acting as stimulators or inhibitors of signal molecule biosynthesis, signal molecule detection, and interference with QS-related phenotypes. Such inhibitors were practically used as research tools for determining the interaction between the QS of pathogens and the host immune system [50]. Results in Table 2 showed the characterized compounds that have quorum quenching properties as previously described. The diterpene alkaloid agelasine that was isolated from the marine sponge *Agelas nakamurai* could inhibit planktonic growth of *Staphylococcus epidermidis* by possessing inhibitory effects against Na^+^, K^+^, and ATPase but did not inhibit biofilm formation [51]. Berberine is an isoquinoline alkaloid with a phytochemical origin that showed a significant negative effect on the growth of *Escherichia coli* by inhibiting the QS system. It mainly acts on the synthesis stage of AI-2 signaling molecules, thereby reducing biofilm formation. The drug-resistant *E.coli* treated with berberine showed a decrease in adhesion thus reducing the pathogenicity [52]. Similarly, the ability of berberine to inhibit violacein pigment in *Chromobacterium violaceum* and in disrupting motility, biofilms, efflux pumps, and eDNA of *P. aeruginosa* were also reported [53]. The decrease in pyocyanin production may be due to berberine; a quinolone analog. This confirms the participation of quinolone compounds in the transcription process of phenazine since quinolone derivatives are typical inducers for the PQS system. In silico studies, berberine could interact with the QS signal receptors of lasR and rhlR. A dihydrochalcone polyphenol known as phloretin serves as a natural plant defense agent. The swarming and swimming of *P. syringae* pv. tomato (Pto) strain DC3000 were reported to be significantly reduced by phloretin whereas AHL production was reduced by 100% at 0.4 mM phloretin [54]. It has been demonstrated that phloretin also can bind to lasR and rhlR, thus favorably controlling the expression of QS-related genes [55].

Flavonoid potency inhibits lasR/rhlR due to their antagonistic effects on the auto-inducer binding receptors thus suppressing the quorum sensing system by employing two hydroxyl moieties in the flavone structure. Thus, treating *P. aeruginosa* cells with flavonoids alters the transcription of QS promoters and suppresses the virulence factor of pathogenicity [54]. Fucoxanthin (FUC) are natural xanthophyll pigments belonging to carotenoids produced by bacteria and microalgae. It can be triggered by the inhibition of biofilm matrix formation and decreasing cell adhesion through the blocking of quorum sensing [56].

One strategy of QSI drugs is degrading AIs through enzymes including lactonases, acylases, and oxidoreductase, thereby preventing the QS systems activation. Most QS inhibitors are similar to autoinducers in the chemical structure, thus they may induce many conformational changes in LuxR-type receptors that impair their binding with RNA polymerase and downregulate the transcription efficiency. This could explain the decline in the expression of QS genes after exposure to a sub-lethal dose of gamma irradiation or sub-inhibitory concentrations of bioactive compounds. Similarly, it was found the binding of lasR with V-06-018, (a small QSI) directly interferes with the native ligand-binding sites in lasR and stabilizes this inactive interaction, thereby preventing dimerization and binding with DNA [57]. To inhibit the binding between odDHL and lasR of *P. aeruginosa*, several novel QSI compounds were created by replacing the lactone ring of odDHL with the pyrone ring or altering the length of the alkyl chain [58]. The docking results provided evidence that H-bonding and hydrophobic interactions with amino acid residues are crucial for understanding the molecular mechanisms involved in the QSI activity of pyridoxal lactohydrazone [59]. The π−π interaction between the pyridine ring and the phenyl ring of tyrosine enhanced hydrazone-lasR complex stability which leads to more inhibitory activity. In addition, the presence of the amino group in the side chain of synthesized QSI compounds plays an important role in inhibitory activity through interaction with threonine in LasR [33]. i.e. the presence of nitrogen atoms and hydroxyl groups increases the QSIs activity by interacting with the amino acid residues in lasR. It's interesting to note that *P. aeruginosa* makes the "orphan" LuxR-type receptor QscR, which may sequester both lasR and rhlR by forming inactive heterodimers. Furthermore, t it is challenging to predict the potential interaction sites for QSI because the rhlR crystal structure is unavailable [44].

## Conclusion

The variation in the virulence phenotyping of *P. aeruginosa* strains under investigation, and the expression of AHL-mediated QS genes that code virulence factors could be explained by the crosslinked network of QS; this means the inhibition in any channel is compensated by activating another but not in the same degree i.e. complete blocking isn’t easy but an efficient inhibition is very possible. Therefore, targeting the QS system to treat bacterial infections provides a new direction for effectively slowing down the development of bacterial resistance toward antibiotics and paving the way for describing quorum-quenching drugs with antibiotics as a successful treatment in MDRP infection. Alternatively, a low irradiation dose of gamma rays instead of higher ones would be applicable to reduce the pathogenicity of MDR microbes that exist in medical tools and products for financial purposes.

### Supplementary Information

**Additional file 1: Table S1.** Evaluation of the Minimum inhibitory concentrations of various halo-bacterial metabolites against selected *P. aeruginosa *strains. **Table S2.** Primers of genes involved in AHLs-quorum sensing. **Fig. S3.** Uncropped and unlabeled agarose gel electrophoresis for PCR products.

## Data Availability

The datasets analyzed during the current study are available from the corresponding author on reasonable request.

## Abbreviations

AIs: Auto-inducers;
AHL: n-acyl homoserine lactones
BHL or C4-HSL: n-butyryl-l-homoserine lactone
Cyclic-di-GMP: Cyclic diguanylate
ESI: Electrospray ionization
ESKAPE pathogens: (*Enterococcus faecium*, *Staphylococcus aureus*, *Klebsiella pneumoniae*, *Acinetobacter baumannii*, *P. aeruginosa*, And *Enterobacter *spp)
FUC: Fucoxanthin
las, rhl, Iqs, and Pqs: The main QS channels
LC–ESI–MS/MS: Liquid chromatography– ionization–tandem mass spectrometry
IQS & PQS: Non-AHL-mediated QS signaling
MDRP: Multi-drug-resistant pathogens
MvfR: Multiple virulence factor regulator
odDHL or 3O-C12-HSL: n-(3-oxododecanoyl)-l-homoserine lactone
PCA: Phenazine-1-carboxylic acid
PCR: Polymerase chain reaction
phz1 and phz2: Identical operons shared in phenazine synthesis
PhzM and PhzS: Two modifying enzymes shared in phenazine synthesis
pqsABCDE: Operon encoding the enzymes involved in the synthesis of non-AHL-mediated QS autoinducers
PvdI, PvdII, and PvdIII: Three classes of pyoverdine
QS: Quorum sensing
QSI: Quorum-sensing inhibitors
ROS: Reactive oxygen species
Rsal: The expression of a transcription repressor of lasI
