# Supplementary material for: Quenching of quorum sensing in multi-drug resistant Pseudomonas aeruginosa: insights on halo-bacterial metabolites and gamma irradiation as channels inhibitors
Source: Ann Clin Microbiol Antimicrob. 2024 Apr 10;23:31. doi: 10.1186/s12941-024-00684-5 (PMC11007959; doi:10.1186/s12941-024-00684-5)
Supplement: Supplementary file 1 — Additional file 1: Table S1. Evaluation of the Minimum inhibitory concentrations of various halo-bacterial metabolites against selected P. aeruginosa strains. Table S2. Primers of genes involved in AHLs-quorum sensing. Fig. S3. Uncropped and unlabeled agarose gel electrophoresis for PCR products. [file 12941_2024_684_MOESM1_ESM.docx]

**Additional file**

**Quenching of quorum sensing in multi-drug resistant *Pseudomonas aeruginosa*: Insights on halo-bacterial metabolites and gamma irradiation as channels inhibitors**

**Reham Talaat^1^, Mohamed N. Abu el‑naga^1^, Heba Abd‑Alla El‑Bialy^1*^, Mohie Z. El-Fouly^1^, Mohamed A. Abouzeid^2-3^**

| **Selected *P. aeruginosa strain*** | **The minimum inhibitory conc. of bio-active metabolites** **(µg/ml)** | |
| --- | --- | --- |
| *P. aeruginosa U3* | 125 (*Halomonas cupida* Halo-Rt1) | 62.5 (*Halomonas elongate* Halo-Rt2) |
| *P. aeruginosa* NCR-RT1 | 250 (*Halomonas cupida* Halo-Rt1) | 7.81 (*Vigibacillus natechei* Halo-Rt3) |
| *P. aeruginosa* NCR-RT2 | 125 (*Halomonas elongate* Halo-Rt2) | 125 (*Sediminibacillus terrae* Halo-Rt4) |
| *P. aeruginosa* NCR-RT3 | 125 (*Halomonas cupida* Halo-Rt1) | 125 (*Halomonas almeriensis* Halo-Rt5) |

**Table (S1):** **Evaluation of the Minimum inhibitory concentrations of various halo-bacterial metabolites against selected *P. aeruginosa* strains**.

**Table (S2): Primers of genes involved in AHLs-quorum sensing**

| **Intact gene amplicons** | **GENE** | **Forward primers** | **Reverse primer** |
| --- | --- | --- | --- |
|  | **LasR** | **5’-ATGGCCTTGGTTGACGGTT-3’** | **5’-GCAAGATCAGAGAGTAATAAGACCCA-3’** |
|  | **rhlR** | **5’-CAATGAGGAATGACGGAGGC-3’** | **5’-GCTTCAGATGAGGCCCAGC-3’** |
| **Internal gene amplicons** | **LasI** | **5’-TCGACGAGATGGAAATCGATG-3’** | **5’-GCTCGATGCCGATCTTCAG-3’** |
|  | **LasR** | **5’-TGCCGATTTTCTGGGAACC-3’** | **5’-CCGCCGAATATTTCCCATATG-3’** |
|  | **rhlI** | **5’-CGAATTGCTCTCTGAATCGCT-3’** | **5’-GGCTCATGGCGACGATGTA-3’** |
|  | **rhlR** | **5’-TCGATTACTACGCCTATGGCG-3’** | **5’-TTCCAGAGCATCCGGCTCT-3’** |
|  | **rhlR**  **(REH)** | **5’-TGAGGAATGACGGAGGCTTT-3’** | **5’-CTCCAGACCACCATTTCCGA-3’** |
| **Ref. genes** | **rpoD** | **5’-GGGCGAAGAAGGAAATGGTC -3’** | **5’-CAGGTGGCGTAGGTGGAGAA -3’** |
|  | **ampC** | **5’-AGATTCCCCTGCCTGTGC -3’** | **5’-GGCGGTGAAGGTCTTGCT -3’** |


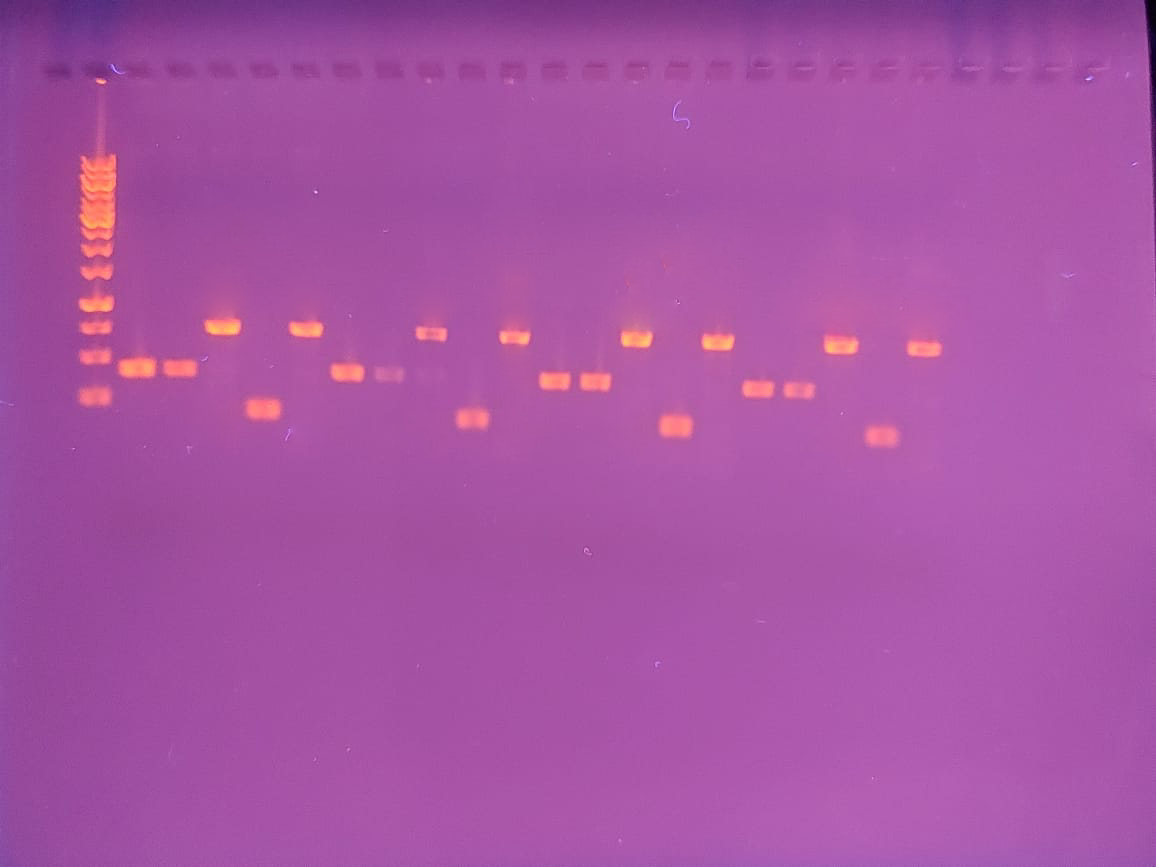


**Fig. (S3): Uncropped and unlabeled agarose gel electrophoresis for PCR products**
